# Dose Dependent Side Effect of Superparamagnetic Iron Oxide Nanoparticle Labeling on Cell Motility in Two Fetal Stem Cell Populations

**DOI:** 10.1371/journal.pone.0078435

**Published:** 2013-11-07

**Authors:** Valentina Diana, Patrizia Bossolasco, Davide Moscatelli, Vincenzo Silani, Lidia Cova

**Affiliations:** 1 Department of Neurology and Laboratory of Neuroscience, IRCCS Istituto Auxologico Italiano, Cusano Milanino, Italy; 2 Department of Chimica Materiali e Ingegneria Chimica G. Natta, Politecnico di Milano, Milan, Italy; 3 Department of Fisiopatologia Medico-Chirurgica e dei Trapianti, “Dino Ferrari” Center, Università degli Studi di Milano, Milan, Italy; Rutgers - New Jersey Medical School, United States of America

## Abstract

Multipotent stem cells (SCs) could substitute damaged cells and also rescue degeneration through the secretion of trophic factors able to activate the endogenous SC compartment. Therefore, fetal SCs, characterized by high proliferation rate and devoid of ethical concern, appear promising candidate, particularly for the treatment of neurodegenerative diseases. Super Paramagnetic Iron Oxide nanoparticles (SPIOn), routinely used for pre-clinical cell imaging and already approved for clinical practice, allow tracking of transplanted SCs and characterization of their fate within the host tissue, when combined with Magnetic Resonance Imaging (MRI). In this work we investigated how SPIOn could influence cell migration after internalization in two fetal SC populations: human amniotic fluid and chorial villi SCs were labeled with SPIOn and their motility was evaluated. We found that SPIOn loading significantly reduced SC movements without increasing production of Reactive Oxygen Species (ROS). Moreover, motility impairment was directly proportional to the amount of loaded SPIOn while a chemoattractant-induced recovery was obtained by increasing serum levels. Interestingly, the migration rate of SPIOn labeled cells was also significantly influenced by a degenerative surrounding. In conclusion, this work highlights how SPIOn labeling affects SC motility *in vitro* in a dose-dependent manner, shedding the light on an important parameter for the creation of clinical protocols. Establishment of an optimal SPIOn dose that enables both a good visualization of grafted cells by MRI and the physiological migration rate is a main step in order to maximize the effects of SC therapy in both animal models of neurodegeneration and clinical studies.

## Introduction

Nanomedicine has a leading role in pharmaceutical research and development of clinical protocols, mainly in the form of nanoparticle-based delivery systems for drugs and imaging agents, especially in the field of stem cell (SC) therapies [1]. Several functionalized nanoparticle formulations have been proposed for medical applications, but few of them have been approved by the Food and Drug Administration (FDA), mainly because of reproducibility problems and uncertain stability in the long term coupled to the absence of consensus guidelines on the required biological testing [2], [3].

Ferumoxides (a suspension of Super Paramagnetic Iron Oxide nanoparticles (SPIOn)), are (FDA)-approved agents which may be accurately, sensitively and easily detectable by non-invasive Magnetic Resonance Imaging (MRI) to monitor grafted cell distribution over time [4]. SPIOns consist of a coated iron oxide core with an overall size greater than 50 nm (coating included) and could potentially be modified for the creation of a personalized nanomedicine tailored to patient- and disease-specific needs [5]. Several reports have demonstrated the safety and reliability of SPIOn labeling as a contrast agent transfer for SC imaging/tracking [6] without apparent side effects on their stemness (as reported by Balakumaran et al. [7] for bone marrow mesenchymal cells). Nevertheless, an increasing number of recent papers are challenging this perspective [8]. U.S. and European governments are also promoting study programs on the impact of nanotechnology and the potential risks of nanoparticles (United States Enviromental Protection Agency (EPA), Nanotechnology & Nanomaterials Research, http://www.epa.gov/nanoscience/index.htm).

SPIOn molecular interactions may exert metabolic or mutagenic effects on the surroundings, especially in the long term, limiting their diagnostic and therapeutic potential [9]. A better understanding of the behavior, collateral effects and toxicity of SPIOn in complex biological fluids/conditions is therefore needed.

Alterations in migration capability are primarily involved in pathological conditions (i.e. metastatic cancers, [10]) and are essential in regenerative medicine (SC therapy, [11]).

Cell movements are finely regulated by Reactive Oxygen Species (ROS) [12] which also play a pivotal role in maintaining SC multipotentiality as well as in the progression of SC-associated diseases [13], [14] and/or cancer [15]. In the present study we analyzed the possible interactions between (dextran-coated) SPIOn loading, migration capability and time course production of ROS in two fetal SC populations, naïve human chorial villi- (hCVCs, collected between 10–12^th^ weeks of pregnancy) and amniotic fluid- (hAFCs, normally harvested around 15^th^ weeks of pregnancy) derived cells. hCVCs and hAFCs, conversely to embryonic SCs, do not raise special ethical concerns. If compared to adult SCs, they display higher multipotentiality and proliferative capabilities, a low immunogenicity as well as an easy accessibility. Moreover, they can be expanded in the long term without tumorigenic risk [16]. These fetal SCs constitute alternative interesting sources for cell therapies in neurodegenerative diseases. As a matter of fact, the neurorescue potential and characteristics of these fetal SCs have been extensively characterized by our group [17].

Herein, we report that SPIOn loading, dose-dependently, affect migration capability, but not ROS production in two fetal SC populations, thus enlightening novel side effects of SPIOn labeling and potential caveats for SPIOn application to (pre)clinical therapy.

## Materials and Methods

### Cell Collection, Culture and Labeling

hAFCs and hCVCs were collected from amniotic fluid or chorionic villi of pregnant women respectively, after specific written informed consent for this research (approved by Institutional Review Board and Ethical Committee of IRCCS-Istituto Auxologico Italiano as 23C106, NP-FSC on April, 12, 2011, following the Italian Law on Stem Cell Research, Senato della Repubblica, Risoluzione (6-00004) (19 luglio 2006) n. 4).

Cells were isolated and grown in Amniomax II (Invitrogen, Carlsbad, CA, USA) at 37°C and 5% CO_2_ in a fully humidified atmosphere for one week before fetal karyotypization by standard procedures (QFQ banding technique) [18], [19]. After cytogenetic analysis, exceeding cultures from pregnancies with a normal fetal karyotype were used for research purpose, as specified in the informed consent. To overcome any possible age-, donor- or sex-related variation, we pooled together 4 different fetal donors (2 with a 46,XX and 2 with 46,XY karyotype) from women between 15–17^th^ gestational week for hAFCs and 4 (2 with a 46,XX and 2 with 46,XY karyotype) from women between 11–13^th^ gestational weeks for hCVCs.

Cells obtained from every sample were harvested after gentle trypsinization, centrifuged at 1,000×g for 10 minutes, pooled together and counted. Dividing cells in suspension were collected as well. Pools were then frozen (600,000 cells/aliquot) and stored in liquid nitrogen. A periodic monitoring of karyotype was conducted to check if subculturing has altered chromosome constitution. hAFCs and hCVCs were loaded with the commercially available MRI agent, dextran coated SPIOn (Endorem®; AMI-25; 11,2 mg Fe/ml, Guerbet, Roissy, France, gently provided by Dr. Bigini) coupled to Poly-L.Lysine (PLL, Sigma Aldrich St. Louis, MO, USA) in Amniomax II, to maximize cell internalization, as already described [20]. In this study, different concentrations and different incubation times were chosen, as specified below for each experiment while PLL concentration was always maintained constant (see Table S1 in File S1).

To specifically verify SPIOn influence on the main biological processes (proliferation, metabolism and migration) which affect the therapeutic potential of SCs we sequentially analyzed them.

### Proliferation Assays

**Figure 1:**
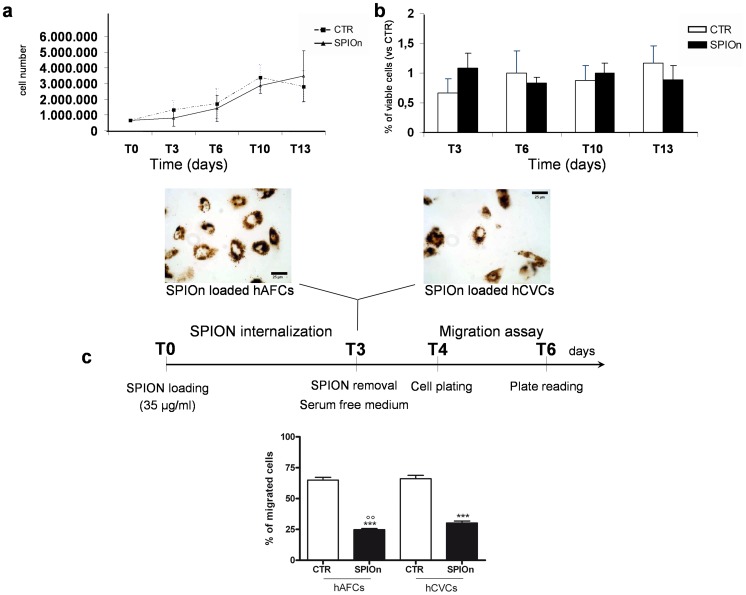
Evaluation of SPIOn labeled cell biological properties and migration. **a** Proliferative and **b** metabolic rates of hCVCs appeared not affected by SPIOn presence, as previously demonstrated by our group for hAFCs [20]; **c** hAFCs and hCVCs were incubated for 72 hours with SPIOn (35 µg/ml) and then migration assay was performed. Data are expressed as a percentage of migrated cells. ***p<0.001 versus respective (unlabeled, control cells, CTR); °°p<0.01 versus SPIOn labeled hCVCs. One way analysis of variance (ANOVA) as specified in the statistical section.

Cell proliferation was performed on 75,000 cells/well in untreated 12-wells plate (NUNC) and each sample was tested in quadruplicate. Cells after SPIOn loading for 72 hrs, were cultured for around 3 weeks, with weekly medium change. At different time points, as detailed in the corresponding graphic, cells were detached and counted (Fig. 1a). Briefly, cells were centrifuged at 1,000×g for 10 min and suspended in 500 µl of fresh medium. Cell viability was assayed at each experimental time point using the Trypan blue exclusion method (Sigma-Aldrich) on an aliquot (10 µl) of each sample.

### Assay of Cell Metabolism

Cell metabolism is deeply influenced by intra-cellular and extra-cellular conditions with potential implications on cell migration, therefore we tested if our experimental conditions (presence of increasing SPIOn doses or 6-hydroxydopamine (6-OHDA)) may affect this biological parameter.

#### Basal metabolism

Cell viability was evaluated by (3-(4,5-dimethylthiazol-2-yl)-5-(3-carboxymethoxyphenyl)-2-(4-sulfophenyl)-2H-tetrazolium, inner salt) MTS assay (CellTilter 96® AQueous Assay, Promega, Madison, WI, USA) following manufacturer’s instructions. To test the potential toxicity of SPIOn, first hAFCs and hCVCs were loaded with increasing concentrations of SPIOn (0 (control, CTR), 5, 10, 25 and 35 µg/ml) for 72 hours, as detailed in the corresponding figure. Cells were then collected and plated (50,000 cells/well) in 96-well plates (NUNC). One hour after a resting time at 37°C, MTS solution (20 µl/well) was added and cells were incubated for another hour at 37°C. The supernatant of cell cultures was collected, centrifuged at 2,000×g for 5 minutes and read at 490 nm with a microplate reader (Elx800, Bio-Tek Instruments Inc., Winooski, VT, USA). Results were expressed as a percentage of viable cells versus CTR (unlabeled cells) using the following function: “labeled cells value”×100÷“unlabeled cells value”.

#### Effect of 6-OHDA exposure on metabolism

Toxicity of 6-OHDA was also assessed on both unlabeled and SPIOn labeled (35 µg/ml, 72 hours of incubation) hAFCs and hCVCs (50,000 cells/well in 96-well plates, NUNC). SH-SY5Y was used as positive control: this is a cell line subcloned from a bone marrow biopsy of a patient affected by a neuroblastoma. SH-SY5Y cells are susceptible to 6-OHDA and therefore represent a good positive control to test the effect of this neurotoxin. Cells were plated and placed at 37°C for 1 hour before being treated with 6-OHDA (100 µM, as previously published, [17]). Survival was measured 1, 4 and 6 hours after exposure to the toxin by adding MTS solution as described above.

Results were expressed as a percentage of viable cells versus CTR (not treated cells), as detailed before.

### Migration Assay

This assay employs a simplified Boyden chamber-like design that consists of two chambers separated by a filter coated with different extra-cellular matrix components. The cell suspension is placed in the top chamber and incubated in the presence of test media eventually containing specific chemoattractants in the bottom chamber. Cells migrate from the top chamber through the coated filter pores to the bottom of the filter. Detection of cell invasion is quantified using calcein acetoxymethyl ester (calcein-AM). Cell dissociation/Calcein-AM solution is placed in the bottom chamber to dissociate the migrating cells from the filter. Calcein-AM is internalized by the cells, and intra-cellular esterases cleave the acetomethylester moiety. Free Calcein fluoresces brightly and is used to quantify the number of cells that have invaded or migrated by comparison with a standard curve.

Migration capability of hAFCs and hCVCs was evaluated by a Cultrex 96 Well Cell Migration Assay kit (Trevigen, Gaithersburg, MD, USA) following manufacturer’s instructions. Briefly, hAFCs and hCVCs were loaded with 0 (control, CTR), 5, 10, 25, 35 µg/ml of SPIOn at time T0 (T indicates here the number of days after SPIOn addition, see Fig. 1c) as described before. Twenty-four hours before migration assay (T3), Amniomax II was replaced with serum free MEM (Gibco Invitrogen). The day after the end of SPIOn incubation (T4 from the beginning of the experiment) hAFCs and hCVCs were collected, resuspended in MEM, plated (50,000 cells/well) in the upper chamber of the specific 96-well plate provided by the manufacturer and placed at 37°C for 48 hours. At T6 migrated cells were quantified as described in manufacturer’s instructions (further details for each experiment design are reported in the corresponding figures). For additional details on all the experimental procedures see Table S2 in File S1.

Additionally in same experiments, to test how hAFC and hCVC migration was influenced by the addition of a chemoattractant (fetal bovine serum (FBS, Sigma Aldrich)), the bottom chamber was loaded with serum free media or with 10, 20 or 30% FBS respectively.

We also mimicked a pathophysiological condition with a well standardized cell model of neurodegeneration to observe any eventual influences of SPIOn presence on cell migration in this experimental setting. Presently, cell models for neurodegenerative diseases, (i.e. Parkinson’s disease (PD)) primarily include neuronal tumor cell lines, such as the human neuroblastoma cells (SH-SY5Y). These cells mimic many aspects of the dopaminergic neuron death observed in PD in a well standardized way, when treated by neurotoxins such as 1-methyl-4-phenyl-pyridinium (MPP+), 6-OHDA, or rotenone without the high variable response of primary dopaminergic cultures derived from different brain regions, after the addition of 6-OHDA [21]. Therefore, in a further experimental setting, we checked if SPIOn labeled cell motility could be affected by the ongoing degeneration of the neighboring cells: we plated the SH-SY5Y cells (50,000 cells/well) cells in the bottom chamber at T3 (as detailed in the corresponding figure and in Table S2 in File S1). The following day (T4), SH-SY5Y cells were challenged with serum free MEM plus 6-OHDA 100 µM dissolved in saline solution containing 0.02% ascorbic acid (both provided by Sigma Aldrich), while unlabeled cells (used as control, CTR) or SPIOn labeled hAFCs and hCVCs were plated in the upper chamber as described before. Migration capability of cells toward the degenerating model was assessed 1, 4 and 6 hours after 6-OHDA exposure. In every experiment, results were expressed as a percentage of migrated cells.

### Staining Procedures for Iron Presence

Evaluation of the internalization of SPIOn in hAFCs and hCVCs was performed by Prussian Blue staining (which labels iron deposits) using a diagnostic kit specific for the detection of ferric iron overload in cells and tissue (PERLS, BioOptica, Milan, Italy). Prussian blue reaction involves the treatment of sections with acid solutions of ferrocyanides. Any ferric ion (+3) present in the tissue combines with the ferrocyanide and results in the formation of a bright blue pigment called Prussian blue, or ferric ferrocyanide. This is one of the most sensitive histochemical tests and will demonstrate even single granules of iron in cells. Labeled cells were rinsed with Phosphate Buffered Saline (PBS, pH 7.2 Sigma Aldrich), collected after trypsinization, plated in 24-well plate, at a density of 30,000 cells/well on glass coverslips and cultured at 37°C with 5% CO_2_ until adhesion. SCs were then fixed with 4% paraformaldehyde (w/v, in 0,1 M PBS) for 20 minutes. Following manufacturer’s instructions, after several washings with distilled water, SCs were incubated 20 minutes with a mixture of reagent A™ and reagent B™ (1∶2.5 v/v in distilled water, provided with the kit) then washed with distilled water. All the passages were done at RT. Coverslips were mounted on microscope slides, observed and images were acquired with a CCD camera, directly connected with a Leica DMRS/HCS (Wetzlar, Germany) microscope.

### Intra-cellular ROS Measurement

For analysis of intra-cellular ROS, hAFCs and hCVCs were labeled with the oxidation-sensitive probe 2′,7′-dichlorodihydrofluorescein diacetate (DAF-FM, Molecular Probes, Invitrogen). Briefly, the day before the experiment, cells were plated in a Fluorometer/Luminometer white 96-well plates (NUNC, Rochester, NY USA; 50,000 cells/well) and left overnight at 37°C to allow adhesion. Cells were then incubated with DAF-FM (5 µM) for 15 minutes in the specific buffer (150 mM NaCl, 3 mM KCl, 20 mM HEPES, 10 mM Glucose, pH 7,4, 2 mM CaCl_2_·6 H_2_O, 1 mM MgCl_2_·6 H_2_O), washed and incubated for 72 hours with SPIOn (35 and 100 µg/ml in buffer). Fluorescence was measured both during incubation with SPIOn (from 1 to 5 hours, two different concentrations 35 and 100 µg/ml; 6–72 hours only 35 µg/ml) and after SPIOn removal (concentration 35 µg/ml, 12, 24, 48 and 72 hours) by a microplate reader (Fluoroskan Ascent FL, Thermo Labsystems, Vantaa, Finland. Reading parameters: ex = 485 nm, em = 530 nm). Thereafter, values were correlated to cell viability measured by digitonin (160 µM, Sigma Aldrich) and propidium iodide (PI, 50 µM, Sigma Aldrich) exposure for 20 minutes at 37°C and subsequent reading with the same instrument (Reading parameters: ex = 530 nm, em = 590 nm) [22]. To avoid misinterpretations, data were normalized on the number of cells in the corresponding well.

### Flow Cytometric Analysis and Fluorescence Imaging

To simultaneously compare their naïve dimension, hCVCs and hAFCs were separately labeled with two different fluorescent dyes (PKH26 Red Fluorescent-, for hCVCs, and PKH67 Green Fluorescent- for hAFCs, Cell Linker Kit; Sigma Aldrich) as previously reported [23]. To confirm our data and to exclude any possible influence of light absorbance/emission, the fluorescent probes were also exchanged between the fetal SC types, using the PKH26 Red linker for hAFCs, and PKH67 Green linker for hCVCs. These fluorescent cell linkers can incorporate a fluorescent dye into lipid regions of the cell membranes, that may be detectable by flow cytometric analysis.

Cells (300,000 cells/sample) were mixed immediately before flow cytometric analysis, but they remained easily distinguishable by their excitation and emission spectra (respectively phycoerythrin and fluorescein). Samples were acquired after lysis by a flow cytometer (FACSCanto II Becton Dickinson) and analyzed using the dedicated Diva software. All possible combinations of the fluorescent dyes were used in order to validate our results in triplicate.

Labeled cells were also observed by fluorescent microscopy to confirm flow cytometry data on a confocal microscope (Nikon Eclipse Ti, Japan).

### Intra-cellular Fe Measurement

Elemental analysis was carried out by inductively coupled plasma optical emission spectrometry (ICP-OES analysis) using an Optima model 8300 instrument (Perkin Elmer, Waltham, MA). Samples were analyzed after digestion using concentrated nitric acid (Sigma Aldrich).

### Statistics

Each experiment was run in triplicate on 5–6 different pools with at least three replicates for each condition and representative values were expressed as mean ± SD.

Data were analyzed using one or two way analysis of variance (ANOVA) followed by Bonferroni *post hoc* test (as specified in the respective figure or table), using a dedicated statistical software (GraphPad Prism, Inc., La Jolla, CA, USA).

## Results

### Preservation of Biological Properties in hCVCs Incubated with SPIOn

Here we demonstrated here that SPIOn internalization (35 µg/ml for 72 hours) did not significantly alter proliferation, metabolism and morphology of hCVCs *in vitro* (Fig. 1 a, b). Indeed, no significant differences were found in the number of cells in culture at different time points (T0, T3, T6 T10 and T13) between loaded and unloaded hCVCs, demonstrating that SPIOn did not altered the cell growth rate (Fig. 1 a). Similarly, the percentage of viable cells was comparable until T13 indicating that metabolic rate of hCVCs appeared not affected by SPIOn presence (Fig. 1 b).

### SPIOn Influenced Migration of hAFCs/hCVCs without Increasing Intra-cellular ROS

In our experimental conditions, we found that hAFCs and hCVCs (labeled with SPIOn 35 µg/ml for 72 hours) did not shown morphological differences, but a significant reduction of migration capability, when compared with unlabeled cells (CTR). Migration rate of loaded cells was approximately halved in respect to CTR (Fig. 1c).

**Figure 2:**
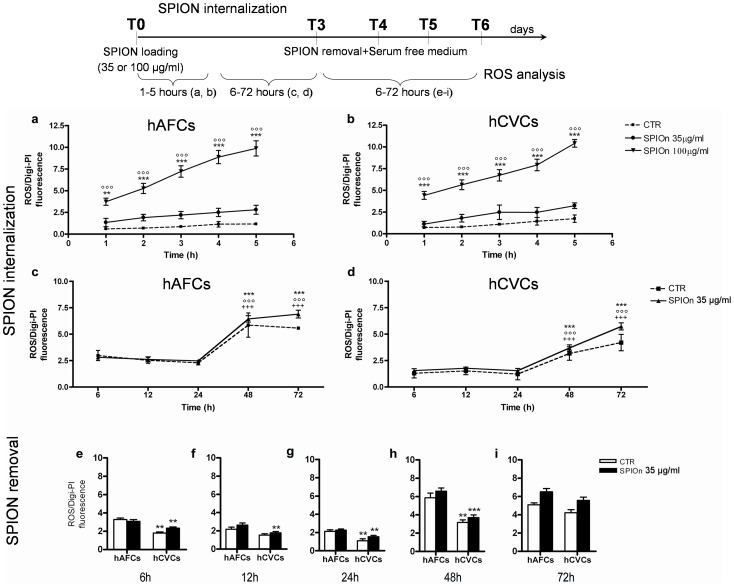
Evaluation of intra-cellular ROS during internalization and subsequent removal of SPIOn. **a** hAFCs and **b** hCVCs were plated and labeled with DAF-FM to visualize intra-cellular ROS, before being incubated with SPIOn (35 or 100 µg/ml). ROS measurement was performed every hour (from 1^st^ to 5^th^) during SPIOn internalization (see experimental scheme). Data are expressed as ROS fluorescent signal normalized for Digitonin-PI. Unlabeled cells represent control condition (CTR). No statistically significant differences were observed between CTR and SPIOn labeled cells. ***p<0.001 versus CTR; °°°p<0.001 versus SPIOn (35 µg/ml). Two way analysis of variance (ANOVA); **c** hAFCs and **d** hCVCs were plated and labeled with DAF-FM and then were incubated with SPIOn (35 µg/ml). ROS evaluation was done at different time (6, 12, 24, 48 and 72 hours) after SPIOn addition. Data are expressed as ROS fluorescent signal normalized for Digitonin-PI. Unlabeled cells represent control condition (CTR). ***p<0.001 versus respective 6 hours; °°°p<0.001 respective 12 hours; +++p<0.001 versus respective 24 hours. Two way analysis of variance (ANOVA) as specified in the Statistical section. To better visualize the significant difference in intra-cellular ROS formation between hAFCs and hCVCs, we compared the two cellular groups at **e** 6, **f** 12, **g** 24, **h** 48 and **i** 72 hours after SPIOn removal. Data are expressed as ROS fluorescent signal normalized for Digitonin-PI. Unlabeled cells represent control condition (CTR). **p<0.01 and ***p<0.001 versus hAFCs. Two way analysis of variance (ANOVA), as specified in the statistical section.

Excess ROS are generated during a variety of cell stresses and may contribute to inflammation and cell/tissue damage. Therefore, to check if SPIOn internalization in cells in these conditions could interfere with physiological cell processes, we measured the ROS formation at different time points during incubation and after SPIOn removal (see experimental scheme Fig. 2). We observed that there were no significant differences in ROS production between unlabeled (CTR) and SPIOn-labeled hAFCs and hCVCs during SPIOn incubation (35 µg/ml from 1 to 5 hours) (Fig. 2a and b, respectively). On the other hand, cells labeled with a maximal dose of SPIOn (100 µg/ml for 72 hours), displayed statistically significant augment of fluorescence intensity at every considered time point if compared to both control (CTR) and 35 µg/ml condition (Fig. 2a and b). Increase of intra-cellular ROS formation is therefore influenced by SPIOn amount.

Between 6 and 24 hours of SPIOn loading (35 µg/ml for 72 hours) the level of intra-cellular ROS was unaffected and comparable between CTR and SPIOn labeled cells for the first 24 hours (Fig. 2c and d). Fluorescence intensity was significantly enhanced at 48 and 72 hours during SPIOn internalization in respect to previous incubation times for both unlabeled-control (CTR) and labeled SCs, but no significant differences were observed between CTR and labeled hAFCs or hCVCs (Fig. 2c and d, respectively). We also checked ROS formation during a time course after SPIOn removal (from 6 to 72 hrs later) in serum-free medium: interestingly, no statistical differences between CTR and SPIOn loaded cells were observed. Moreover, the level of intra-cellular ROS produced by hCVCs was significant lower than hAFCs during the first 48 hrs (Fig. 2 e–h). This difference disappeared 72 hours after SPIOn removal: at this time point fluorescence intensities of the two cell populations were comparable (Fig. 2i).

### Migration Rate of hAFCs an hCVCs is Dose Dependently Influenced by SPIOn Presence

**Figure 3:**
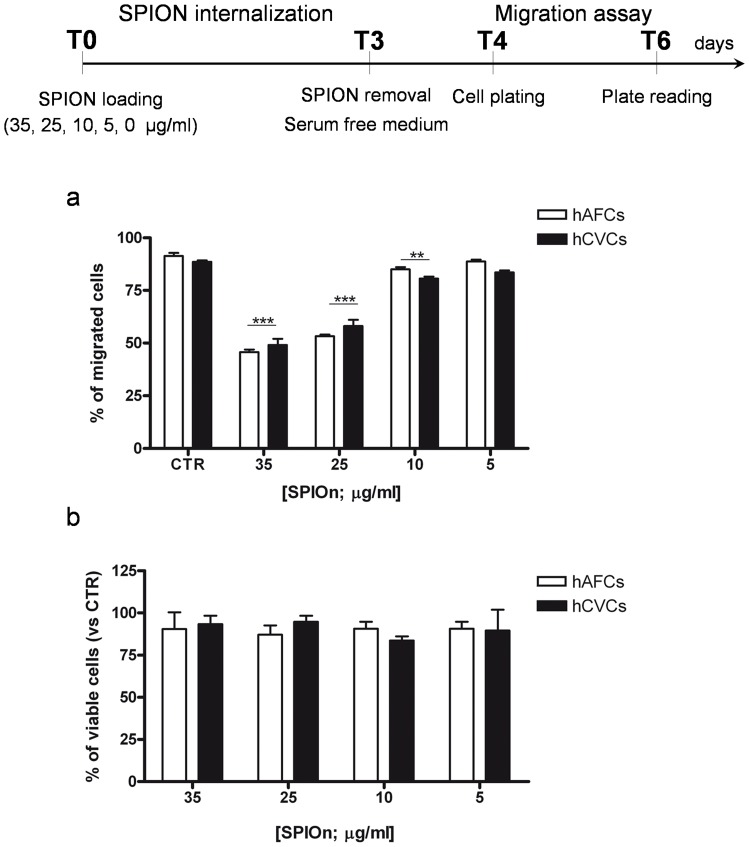
Migration and survival of hAFCs and hCVCs labeled with different SPIOn concentrations. **a** Cells were labeled with different concentration of SPIOn for 72 hours before migration assay. Data are expressed as a percentage of migrated cells. *p<0.05, **p<0.01 and ***p<0.001 versus respective CTR (control, unlabeled cells). Two way analysis of variance (ANOVA); as specified in the statistical section; **b** hAFCs and hCVCs labeled with different SPIOn concentrations were used to test SPIOn toxicity by MTS assay. Data are expressed as a percentage of viable cells versus unlabeled cells used as control (CTR). Two way analysis of variance (ANOVA); as specified in the statistical section.

In a second set of migration experiments, we wanted to verify if impairment of cellular motility in our system was due to the concentration of SPIOn used. The same experiments were performed with decreasing concentration of the nanoparticles (from 35 to 5 µg/ml). We observed a progressive significant recovery of migration, inversely proportional to SPIOn concentration. Graph reported in Fig. 3a clearly illustrates that, starting from SPIOn concentration of 25 µg/ml, there is a significant increase in SPIOn-labeled cell migration, until no more differences were retrieved between unlabeled cells (CTR) and hAFCs or hCVCs labeled with 5 µg/ml SPIOn for 72 hours. Indeed, if compared to unlabeled cells, only about 50% of both SPIOn labeled (35 µg/ml) hCVCs and hAFCs migrated. However, when decreasing the concentration of SPIOn labeling to 5 µg/ml, the rate of migration was completely recovered.

MTS assay was performed to check cell survival under every condition and ascertain that reduced cell migration was not due to hAFC and hCVC death. We observed no significant differences in hAFC and hCVC viability, even when the higher concentration of SPIOn was used (35 µg/ml; Fig. 3b).

### Naïve hAFCs and hCVCs have Similar Dimension and Internalized the same amount of SPIOn

During migration experiments we observed that SPIOn-labeled hAFCs displayed a reduced motility if compared to hCVCs (24.8±5.2 vs 30.2±8.8, °°p<0.01 versus SPIOn labeled hCVCs) in the same experimental conditions (Fig. 1c). This discrepancy could be either due to different cell dimensions or to different SPIOn uptake between the two SC populations, so we verified any eventual variations of these parameters in our experimental settings.

**Figure 4:**
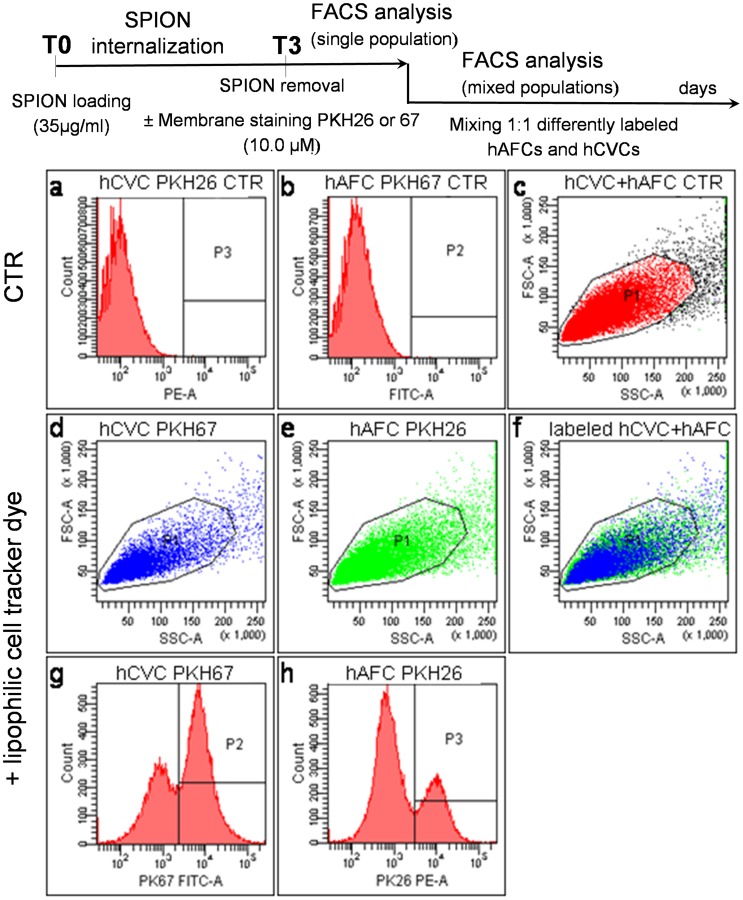
Flow cytometric analysis. Fluorescent cell linker labeled hCVCs (here PKH26) and hAFCs (here PKH67) were analyzed by flow cytometry in order to investigate differences in size and cell complexity. Representative figure of unlabeled hCVCs and hAFCs (**a,b**): when they were mixed, a single cell population was detected (**c**); different labeling of hCVCs (**d,g**) and hAFCs (**e,h**), before mixing the cells, allowed us to identify simultaneously both the cell population confirming their comparable size and complexity (**f**).

Each naïve cell type was loaded with the same amount of SPIOn (35 µg/ml) for 72 hours and separately labeled with a specific fluorescent membrane linker (PKH26 Red for hAFCs and PKH67 Green for hCVCs, then the two linkers were switched between the SC types to validate the retrieved data). The two cell types were then mixed for FACS analysis (see experimental paradigm in Fig. 4) to exclude cell size differences since bigger cells have higher surface, higher iron uptake and then lower migration rate.

No difference between the cell sizes was detected, as shown in the representative graphics of Figure 4 depicting single populations (Fig. 4 a,b CTR; Fig. 4 d,e; Fig. 4 g,h), or analysis on the mixed population (Fig. 4c,f and confocal image in Figure S1). Moreover, we quantified the amount of SPIOn loaded in hAFCs and hCVCs after different internalization times. ICP-OES analysis reported in Table 1 showed that the iron content in both samples (hAFCs and hCVCs) increases during the 72 hours incubation from a negligible amount to a bit more than 1 ppm. Therefore, the time-dependent uptake of SPIOn in our samples directly corresponded to the measurable amount of intra-cellular iron. Moreover, our analysis confirmed that the two cellular types internalized a similar concentration of SPIOn, at every time point considered (24, 48 and 72 hours of SPIOn internalization; see Table 1).

**Table 1:** Intra-cellular ferric ions measurement.

	hAFC	hCVC
**CTR**	0.025±0.01	0.035±0.01
**24 hours**	0.455±0.11	0.550±0.13
**48 hours**	0.550±0.13	0.705±0.16
**72 hours**	1.310±0.30	1.670±0.38

Cells were incubated for 24, 48 or 72 hours with 35 µg/ml of SPIOn. Iron content expressed as Fe and Fe_3_O_4_ in control-samples of AFC, CVC and physiological solution along with a Fe_3_O_4_ standard at 35 ppm are reported. Unlabeled cells represent control condition (CTR). Data (mg/L) are expressed as mean ± SD and analyzed with two way analysis of variance (ANOVA).

Altogether our data demonstrate that the two naïve populations had the same size and iron uptake thus excluding that the retrieved differences in migration between the two cell types are not related to these parameters.

### The Presence of Serum could Partially Restores the Physiological Migration Rate in SPIOn Labeled Cells

**Figure 5:**
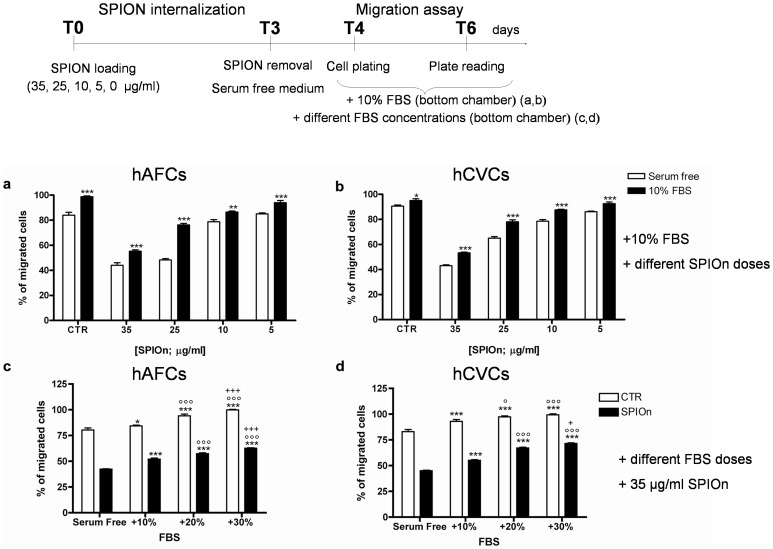
Effect of serum concentration on the migration of SPIOn labeled hAFCs and hCVCs. **a** hAFCs and **b** hCVCs were incubated for 72 hours with different concentration of SPIOn and then migration assay was performed. In these experimental setting, medium with 10% FBS was added in the bottom chamber. Data are expressed as a percentage of migrated cells. Unlabeled cells represent control condition (CTR). **p<0.01 and ***p<0.001 versus serum free medium. Two way analysis of variance (ANOVA) as specified in the Statistical section; **c** hAFCs and **d** hCVCs were labeled with SPIOn 35 µg/ml (72 hours of incubation) and migration assay was performed. In these experiments medium plus 10, 20 or 30% FBS was added in the bottom chamber. Data are expressed as a percentage of migrated cells. Unlabeled cells represent control condition (CTR). *p<0.05 and ***p<0.001 versus serum free medium. °p<0.05 and °°°p<0.001 versus 10% FBS; +p<0.05 and +++p<0.001 versus 20% FBS. Two way analysis of variance (ANOVA) as specified in the statistical section.

To check if in our experimental condition the chemoattractive property of serum is able to restore physiological migratory properties of SPIOn-labeled cells (concentrations from 5 to 35 µg/ml), medium with 10% FBS was added in the bottom chamber, as detailed in the experimental design, Fig. 5. We observed a significant increase of migration rate for both unlabeled (CTR) and SPIOn labeled cells: this effect was detected for both hAFCs and hCVCs independently from SPIOn concentrations (Fig. 5 a and b, respectively). Starting from SPIOn concentrations of 25 µg/ml, comparable migratory properties between CTR and SPIOn loaded cells were retrieved by the addition of 10%FBS.

We also investigated if increasing percentages of FBS (10,20 or 30% FBS) could dose-dependently restore migration in SPIOn-labeled cells (35 µg/ml for 72 hours), as detailed in the experimental design, Fig. 5. A strong enhancement of cellular migration was found in every considered condition, even in CTR samples. Interestingly, both SPIOn labeled hAFCs and hCVCs displayed a significant increase of migrated cells in presence of 30% FBS (Fig. 5c and d, respectively). In this condition indeed, the percentage of SPIOn labeled migrated cells resembled the unlabeled (CTR) one (80%±3.104 CTR and 62%±1 SPIOn loaded hAFCs; 83%±3.062 CTR and 71%±1.741 SPIOn loaded hCVCs).

### Neurodegenerative Surroundings Influence the Migration Rate of SPIOn Labeled Cells

SC rate of migration is strongly influenced by the surroundings: their movements can be stimulated by soluble factors (chemotactic signals) released by damaged cells or tissues. To observe if a lesioned environment may influence migration of SPIOn loaded hAFCs and hCVCs we exploited SH-SY5Y cell line exposed to 6-OHDA, as a well standardized model of cell neurodegeneration.

**Figure 6:**
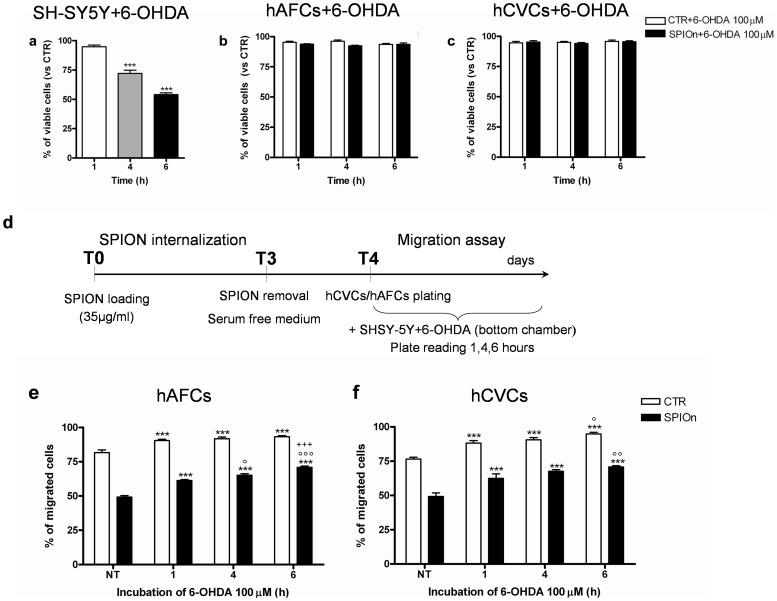
Role of ongoing degeneration on cell migration. **a** SH-SY5Y cells were plated and treated with 6-OHDA 100 µM. After 1, 4 and 6 hours viability was measured by MTS assay. Data are expressed as a percentage of viable cells versus CTR (no 6-OHDA treated cells). ***p<0.001 versus CTR. One way analysis of variance (ANOVA) as specified in the Statistical section; **b** hAFCs and **c** hCVCs were incubated for 72 hours with SPIOn (35 µg/ml) and then were treated with 6-OHDA (100 µM). MTS assay was performed 1, 4 and 6 hours after 6-OHDA/toxin addition. Data are expressed as a percentage of viable cells versus respective CTR (no 6-OHDA treated cells). Two way analysis of variance (ANOVA) as specified in the Statistical section. **d** timeline of migration experiments; **e** hAFCs and **f** hCVCs were labeled with SPIOn 35 µg/ml (72 hours of incubation) followed by the migration assay. In these experiments, SH-SY5Y cells were plated in the bottom chamber and then treated with/exposed to 6-OHDA (100 µM). Migration was evaluated 1, 4 and 6 hours after treatment. Data are expressed as a percentage of migrated cells. Unlabeled cells represent control condition (CTR). ***p<0.001 versus not treated (NT) SH-SY5Y cells; °p<0.05, °°p<0.01 and °°°p<0.001 versus 1 hour treatment; +++p<0.001 versus 4 hours treatment. Two way analysis of variance (ANOVA) as specified in the statistical section.

First we checked the sensitivity to 6-OHDA for all our cell types. SH-SY5Y, hAFCs and hCVCs were plated and treated with 6-OHDA (100 µM). By MTS assay, viability was then tested after 1, 4 or 6 hours of exposition to the toxin. We found that SH-SY5Y survival was significantly reduced starting from 4 hours after 6-OHDA exposure (Fig. 6a). On the other hand, MTS assay performed on 6-OHDA-treated hAFCs and hCVCs revealed that the metabolic rate of these cells remained unaffected by the neurotoxin presence (Fig. 6b and c, respectively).

Thereafter, we performed a migration assay plating SH-SY5Y on the bottom chamber while unlabeled (CTR) and SPIOn-labeled hAFCs and hCVCs were seeded in the upper chamber, as illustrated in Fig. 6d and Table S2. The number of migrated cells measured at different times after SH-SY5Y 6-OHDA exposure revealed that, in presence of ongoing degeneration, SPIOn labeled hAFCs and hCVCs could increase their migration rate (Fig. 6e and f, respectively). Interestingly, migration was time dependently enhanced by SH-SY5Y 6-OHDA treatment in all experimental conditions (Fig. 6e and f).

## Discussion

Molecular medicine imaging is a recent biomedical approach that allows the non invasive *in vivo* characterization of cell biological processes over time, especially in SC therapy [24]. Several subtypes of inorganic nanoparticles have been developed, as a contrast agent, leading to their safe application in different fields, from drug delivery in cancer therapy [25] to (pre) clinical dynamic imaging of transplanted SCs [1]. SPIOn coupled to PLL can be easily and safely internalized in several cell types and tracked in animals by MRI imaging after grafting [20], [26]. Biology and physiology of human embryonic stem cells are not affected by SPIOn labeling (concentration 50 µg/ml for 24 hours) maintaining a persistent signal during long term MRI imaging (21 days) [27].

Recently, it has been reported that implanted SPIOn patches (with an iron concentration of 552 mg/ml) in animals can undergo slow progressive degradation *in vivo* over time [28], thus potentially interfering with cell physiology, mainly in relation to macrophage activation and presence of multinucleated giant cells. A possible pro-inflammatory and allergenic property of SPIOn has been suggested after single intra-tracheal administration of SPIOn at different concentrations in mice [29]. Several additional potential side effects of SPIOn labeling (i.e. membrane leakage of lactate dehydrogenase, impaired mitochondrial function, inflammation, apoptosis and DNA damage) have been summarized by Singh et al [8].

Our data indicate that SPIOn labeling dose-dependently affect human fetal SCs *in vitro* confirming a previous report on altered migration rate of neural progenitor cells loaded with SPIOn *in vivo* [30]. The reported novel collateral *in vitro* effects of SPIOn presence in human fetal SCs may therefore negatively influence the outcomes of SPIOn loaded cells in clinical regenerative protocols.

In this study we used a limited dose of SPIOn (35 µg/ml for 72 hours) that has no collateral effect and is safe on the most important biological properties of hCVCs *in vitro* (as presented here) and hAFCs both *in vitro* and *in vivo* [20]. As a matter of fact, we have previously characterized the stem and therapeutic potentials of both hAFCs and hCVCs [17] analyzing in details the safety, efficiency and reliability of two different tracers (SPIOn and Hoechst 33258) for the imaging of hAFCs *in vitro*, *in vivo* and *ex vivo* [20]. We also observed the *in vivo* survival of SPIOn loaded hAFCs, till 56 days after transplantation, in the lateral ventricles of wobbler mice (an animal model of amyotrophic lateral sclerosis) jointed to the long-term permanence of grafted cells even in damaged areas demonstrating the usability of these SPIOn labeled cells in animal models of neurodegeneration [20].

Nevertheless, we observed an enhanced ROS production, after 48 hours of SPIOn loading at low dose, without reaching statistical significance for both SC types with respect to controls. ROS production was lower in hCVCs than in hAFCs at any time point considered, although comparable cell size and iron uptake were demonstrated in both fetal SCs over time. Moreover, in our experimental conditions, SPIOn loaded hCVCs displayed lower ROS production, at least until 48 hours after SPIOn removal. Similar ROS levels were observed after 72 hours in serum free medium for both SC types, while only 9–12 hours are required in human embryo kidney (HEK 293) and HeLa cervix carcinoma cells to observe major ROS-induced apoptotic cell death triggered by withdrawal of cell survival factors [31]. Concordantly to our data, the level of antioxidant defense necessary for maintaining SC identity appears developmentally regulated and decreases along differentiation [15] since SC metabolism and potential appear directly connected to redox homeostasis [14].

Our data also show that the higher dose of SPIOn loading (100 ug/ml, well known in literature to cause an important increase of ROS formation [32]) produces substantial ROS increases in few hours while only longer SPIOn incubations at low dose (48–72 hours) generate significant augment of intra-cellular ROS levels in fetal SCs in respect to previous incubation times. ROS generation, jointed to decreased cell proliferation, mitochondrial damage, apoptosis and autophagy, were also described after higher SPIOn labeling (>50 µg/ml for 48 hours) in human endothelial cells [33]. In our experimental conditions the significant impaired rate of cell migration in SPIOn loaded cells appears independent from the contemporaneous ROS increase (comparable between control and treated cells), 72 hours after SPIOn removal. Altogether our data, also supported by the literature, suggest that the SPIOn loading time should not exceed the 24 hours to guarantee the lowest ROS production, even at low SPIOn concentration (35 µg/ml).

Migration is an essential process during cell life in organism from development to ageing [34], but even more a well organized collective migration towards damage areas is fundamental for tissue regeneration in cell therapy, as already demonstrated for neurodegenerative diseases and brain tumors [35], [36]. Previous studies have demonstrated that SPIOn labeling may, dose-dependently, affect the intra-cellular actin cytoskeleton and microtubule architecture, until iron content is eluted by cell division [37], [38]. We previously demonstrated by transmission electron microscopy that the SPIOn concentration used here did not alter the cytoplasmic ultrastructure of hAFCs [20]. Nevertheless, here we retrieved a dose-dependently reduced migration rate of both SPIOn labeled SCs. Interestingly, our data showed that migration impairment due to SPIOn loading in SCs may be at least partially recovered by a chemoattractive (increasing FBS doses) or an ongoing degenerative surrounding (such as SH-SY5Y exposed to 6-OHDA [22]) thus demonstrating that the motility remains physiologically influenced by the appropriate stimuli. This observation could therefore explain the qualitatively comparable distribution of control and SPIOn labeled SCs after transplantation [30], even in a neurodegenerative environment [20]. Nevertheless, our data here demonstrates that only the lowest concentration of SPIOn (5 µg/ml) guarantees a migration rate comparable to controls. Concordantly, cell motility of neural SC appeared reduced *in vivo* after SPIOn labeling (25 µg/ml for 24 hours), although a time dependent reduction of iron content by exocytosis partially restored the physiological migration rate [30]. Cell invasion appeared also inhibited post SPIOn internalization in human differentiated cells [39], [40] while we report and quantify here for the first time *in vitro* a reduced migration rate of human SCs. We also speculate that only a careful choice of SPIOn dose and the SC source could guarantee best imaging, physiological cell migration rate and the correct assessment of the eventual therapeutic effects, both in animal models and clinical applications.

The dose-dependent impairment of cell motility we describe here, may therefore affect the outcomes of (pre)-clinical trials of SPIOn loaded SCs, especially in a neurodegenerative/pathological environment where misregulation of metal homeostasis [41] or iron accumulation [42] already play a critical role. A direct link between excessive metal-induced oxidative stress and human diseases have been demonstrated [43] suggesting that iron chelation might be a promising therapeutics, at least in several neurodegenerative diseases [44].

Moreover, diffuse neurotoxicity/multiple side effects of iron nanoparticle have been described in several cell systems from neuronal cell models to Central Nervous System (CNS) microglia and primary neuronal cells. Intra-cellular delivery of even moderate levels of iron oxide nanoparticles (iron concentration from 0.15 to 15 mM) adversely affects cell functions of the rat pheochromocytoma cell line (PC 12), an ideal model system for the study of neural stem cell development and differentiation. Multiple side-effects have been described: i) statistically significant reductions in PC12 cell viability, coupled to significant cell detachment, within the first 48 hours after nanoparticle loading; ii) changes in morphology and cytoskeletal structure of cells after differentiation into neuronal type cells post Nerve Growth Factor exposure (100 ng/ml) with minimal axonal/microtubule sprouting deprived of mature neurites and neuronal branching, dose dependently correlated to the iron content; iii) significantly diminished expression of the neuronal protein growth-associated protein-43 associated with axonal growth, neuronal plasticity, learning and development as well as physiological function of neuronal system [45]. Concordantly, primary cultures of CNS microglial cells have demonstrated clear-cut decreases in cell viability starting from 20 µg/mL concentrations of paramagnetic nanoparticles. Moreover, a differential iron uptake by microglial cells has been observed in comparison to the surrounding brain cells, thus potentially limiting efficient nanoparticle delivery to mixed populations of neural cells, characterized by heterogeneous kinetics of iron loading [46]. Similarly, uptake studies in primary mixed neuronal/glial cultures from cerebellum have revealed predominant uptake of magnetic nanoparticles (at concentration of 50 µg/ml) by microglia and a significant increase in their number. Conversely, in primary mixed Schwann cell/fibroblast cultures it has been showed a comparable iron uptake, but a decrease in the Schwann cell number after nanoparticle loading. Active microglial phagocytosis, due to iron uptake, has been reported in organotypic co-cultures of spinal cord slices and peripheral nerve grafts suggesting also a potential metal-induced immune system reactivity *in vivo* [47]. Viability of cultured microglial cells and alterations in the membrane integrity have been described compromised by 3 hour incubations with iron concentrations of 450 or 1500 µM while the simple coating material, in the absence of the iron oxide core, has not effect on cell survival [48].

Our data may be also useful to efficiently calibrate the best SPIOn content and incubation time to maximize beneficial effects in cells preserving best imaging resolution, especially for novel therapeutic approaches, such as the application of magnetic iron nanoparticles to reduce the amyloid aggregates typical of Parkinson’s, Alzheimer’s Disease and type II diabetes [49]. The reported altered migration of SPIOn loaded SCs and the recovery in a neurodegenerative environment appears particularly critical in neurological diseases where tissue accessibility to the damaged area could be precluded or harmful and therefore neurorescue requires active migration.

## Conclusion

Our data enlighten novel collateral effects of SPIOn presence in human fetal SCs *in vitro* which may interfere with the outcomes of SC grafts: 1) long-term increase of ROS (over 24 hours incubation time) independently from SPIOn presence; 2) dose-dependent impairment of migration in SPIOn loaded cells which appears correlated to the SC type used in the study. However we also detected a partial recovery of cell motility, dose-dependently, by a chemoattractant (here FBS) or, time dependently, by a degenerative surrounding (here SH-SY5Y exposed to 6-OHDA).

Our results also suggest that SC SPIOn labeling is a promising strategy, but requires future experiments and optimization *in vivo* for its safe application to nanomedicine and nanodiagnostic, especially for the treatment of neurodegenerative diseases.

## Supporting Information

Figure S1 **Representative confocal image of PKH26-labeled hCVCs (red) and PKH67-labeled hAFCs (green) showing comparable cell size between the two populations, thus supporting the flow cytometry data.** (Figure 4).
(TIF)

File S1 **This file contains Tables S1 and S2.** Table S1: Scheme of experiment for SPIOn labeling. Table S2: Experimental protocol for migration assay.
(DOC)
