# Supplementary figures and images for: Dose Dependent Side Effect of Superparamagnetic Iron Oxide Nanoparticle Labeling on Cell Motility in Two Fetal Stem Cell Populations
Source: PLoS One. 2013 Nov 7;8(11):e78435. doi: 10.1371/journal.pone.0078435 (PMC3820601; doi:10.1371/journal.pone.0078435)

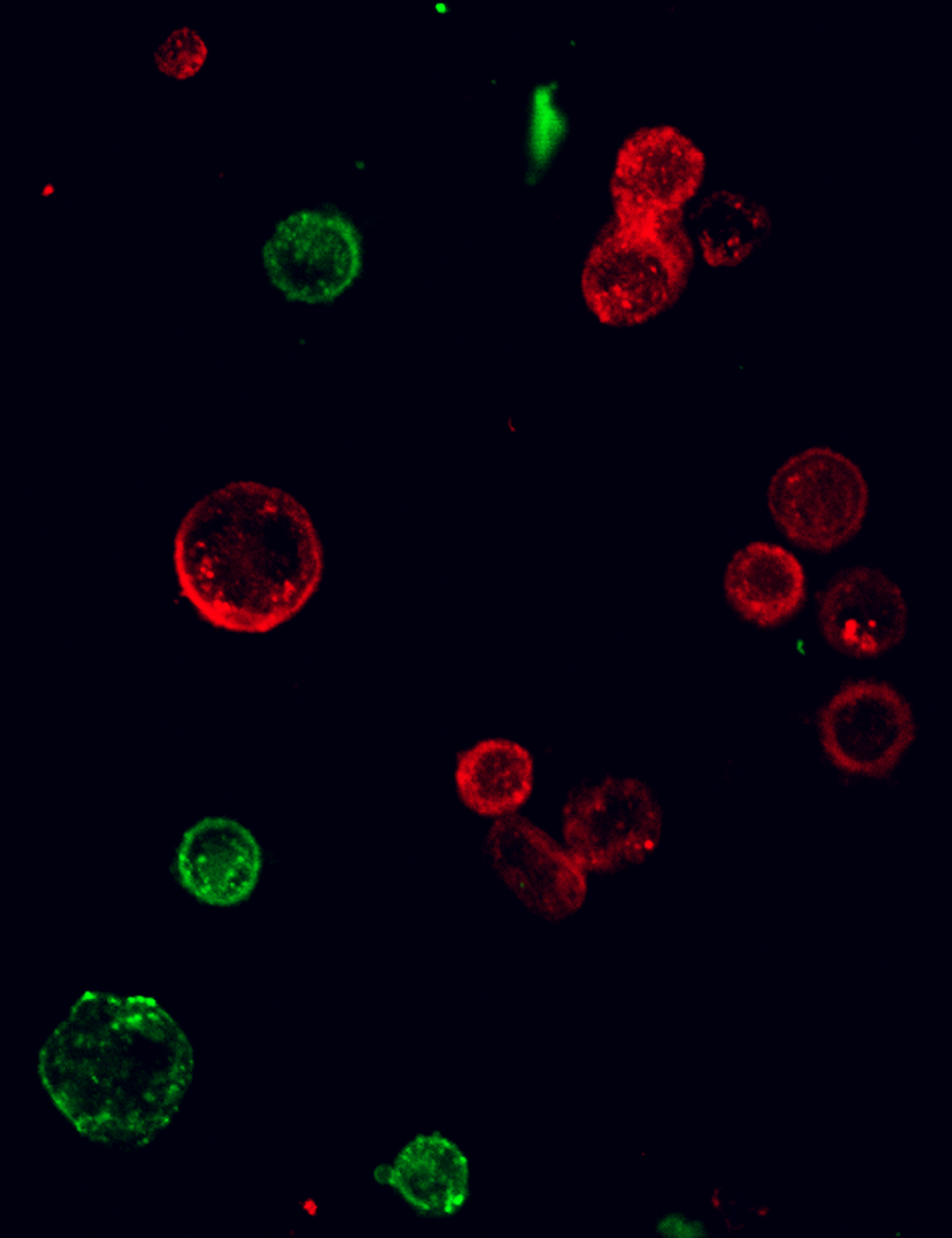

Supplement: Figure S1 — Representative confocal image of PKH26-labeled hCVCs (red) and PKH67-labeled hAFCs (green) showing comparable cell size between the two populations, thus supporting the flow cytometry data. (Figure 4). (TIF) [file pone.0078435.s001.tif]
