# Supplementary material for: Dose Dependent Side Effect of Superparamagnetic Iron Oxide Nanoparticle Labeling on Cell Motility in Two Fetal Stem Cell Populations
Source: PLoS One. 2013 Nov 7;8(11):e78435. doi: 10.1371/journal.pone.0078435 (PMC3820601; doi:10.1371/journal.pone.0078435)
Supplement: File S1 — This file contains Tables S1 and S2. Table S1: Scheme of experiment for SPIOn labeling. Table S2: Experimental protocol for migration assay. (DOC) [file pone.0078435.s002.doc]

Table S1

| **Step 1 :** | **Step 2 :** | **Step 3 :** | **Step 4 :** | **Step 5 :** |
| --- | --- | --- | --- | --- |
| Cell culture of hCVCs and hAFCs in single samples | Pooling of cells:  4 single samples  (2 samples 46,XY + 2 samples 46,XX for each pool) | Freezing of the pools in liquid nitrogen (Aliquots of 600,000 cells) | Defrosting of the pools | Loading of dextran coated SPIOn+PLL (0.4 mg/ml) |

Table S2

**MIGRATION ASSAY - kit**

UPPER CHAMBER:

BOTTOM CHAMBER

The 2 chambers are separated by a membrane which is cell permeable

|  | **Migration capability of hAFCs and hCVCs** | **Influence of serum on migration** | **Influence of ongoing degeneration on migration** |
| --- | --- | --- | --- |
| **T0** | Loading of hCVCs and hAFCs with SPIOn  (0, 5, 10, 25, 35 µg/ml) +PLL | Loading of hCVCs and hAFCs with SPIOn  (0, 5, 10, 25, 35 µg/ml) +PLL | Loading of hCVCs and hAFCs with SPIOn  (0, 5, 10, 25, 35 µg/ml) +PLL |
| **T3** | Change of culture medium with fresh FBS-free medium | Change of culture medium with fresh FBS-free medium | Change of culture medium with fresh FBS-free medium  Bottom chamber: plating of SH-SY5Y cells |
| **T4** | Upper chamber: cell plating with fresh FBS-free medium  Bottom chamber: fresh FBS-free medium | Upper chamber: cell plating with fresh FBS-free medium  Bottom chamber: fresh medium + (0, 10, 20 or 30%) FBS | Upper chamber: cell plating with fresh FBS-free medium  Bottom chamber: change of medium with fresh FBS-free medium + 6OHDA 100µM |
| **+ 1 hour** | - | - | Calcein-AM addition +Plate reading |
| **+ 4 hours** | - | - | Calcein-AM addition +Plate reading |
| **+ 6 hours** | - | - | Calcein-AM addition +Plate reading |
| **T6** | Calcein-AM addition +Plate reading | Calcein-AM addition +Plate reading |  |
